# Bibliographical

**Published:** 1879-12

**Authors:** 


### BIBLIOGRAPHICAL.

### THE DOSE BOOK.

*By C. H. Leonard, M. A., M. D., Detroit, Michigan.*

The third edition of this work revised and enlarged, has just been issued. It contains the doses of over two thousand five hundred preparations, among which are all the leading therapeutic agents, with a large number of those that are less frequently indicated, still are very important. More than two hundred preparations have been added to the list in this edition. The importance of this is quite apparent when it is remembered that new therapeutic agents are being introduced almost daily. The aim has been to make the work most complete; the utmost care has been exercised to secure the greatest freedom from errors in doses and orthography. The work is a valuable one to the physician, and particularly to the specialist, of whom it may with propriety be said, that he is necessarily less familiar with doses and prescriptions, than the general practitioner, so that to him such a work becomes the more a necessity.

---

The Physicians Visiting List is just issued by Lindsay & Blakiston. It contains quite a variety of useful information, viz: a list of poisons and the antidotes; the metric system for prescriptions; the doses of all the common remedies expressed in the common, and also in the metric terms.

The arrangement of the engagement and memorandum pages is excellent for the physician, and may be used by the dentist as well. For the use of the latter it is about as well adapted as any special book that has as yet appeared.

